# *Eucommia ulmoides* Leaves Alleviate Cognitive Dysfunction in Dextran Sulfate Sodium (DSS)-Induced Colitis Mice through Regulating JNK/TLR4 Signaling Pathway

**DOI:** 10.3390/ijms25074063

**Published:** 2024-04-05

**Authors:** Han Su Lee, Jong Min Kim, Hyo Lim Lee, Min Ji Go, Dong Yeol Lee, Chul-Woo Kim, Hyun-Jin Kim, Ho Jin Heo

**Affiliations:** 1 Division of Applied Life Science (BK21), Institute of Agriculture and Life Science, Gyeongsang National University, Jinju 52828, Republic of Korea; ns3005@naver.com (H.S.L.); myrock201@gnu.ac.kr (J.M.K.); gyfla059@gnu.ac.kr (H.L.L.); rh9245@naver.com (M.J.G.); hyunjkim@gnu.ac.kr (H.-J.K.); 2 Research & Development Team, Gyeongnam Anti-Aging Research Institute, Sancheong 52215, Republic of Korea; dylee1984@gari.or.kr; 3 Division of special Forest Resources, Department of Forest Bio-Resources, National Institute of Forest Science, Seoul 02455, Republic of Korea; futuretree@korea.kr

**Keywords:** *Eucommia ulmoides*, intestinal function, inflammation, cholinergic system

## Abstract

Ulcerative colitis (UC) is one of the inflammatory bowel diseases (IBD) that is characterized by systemic immune system activation. This study was performed to assess the alleviative effect of administering an aqueous extract of *Eucommia ulmoides* leaves (AEEL) on cognitive dysfunction in mice with dextran sulfate sodium (DSS)-induced colitis. The major bioactive compounds of AEEL were identified as a quinic acid derivative, caffeic acid-O-hexoside, and 3-O-caffeoylquinic acid using UPLC Q-TOF/MS^E^. AEEL administration alleviated colitis symptoms, which are bodyweight change and colon shortening. Moreover, AEEL administration protected intestinal barrier integrity by increasing the tight junction protein expression levels in colon tissues. Likewise, AEEL improved behavioral dysfunction in the Y-maze, passive avoidance, and Morris water maze tests. Additionally, AEEL improved short-chain fatty acid (SCFA) content in the feces of DSS-induced mice. In addition, AEEL improved damaged cholinergic systems in brain tissue and damaged mitochondrial and antioxidant functions in colon and brain tissues caused by DSS. Also, AEEL protected against DSS-induced cytotoxicity and inflammation in colon and brain tissues by c-Jun N-terminal kinase (JNK) and the toll-like receptor 4 (TLR4) signaling pathway. Therefore, these results suggest that AEEL is a natural material that alleviates DSS-induced cognitive dysfunction with the modulation of gut–brain interaction.

## 1. Introduction

Ulcerative colitis (UC) is a disease that belongs to a subcategory of inflammatory bowel disease (IBD) and is characterized by inflammation starting from the rectum and causing inflammation throughout the colon [1]. Clinical symptoms include abdominal pain, diarrhea, bloody stools, and weight loss, and it is known to be caused by a combination of various causes such as genetics and environmental factors and gut microbiota dysbiosis, but the exact cause is still unclear [2]. One of the known forms of pathogenesis is that mucus produced from goblet cells among the intestinal epithelial cells (IECs) in the intestinal lumen forms a mucus layer covering the intestinal epithelial layer, and the mucus layer of the intestinal barrier is damaged due to complex causes [3]. Afterward, when the intestinal epithelium is directly exposed to external antigens, the tight junctions (TJs) between the IECs are damaged, and intestinal permeability increases [4]. When intestinal permeability increases, external antigens enter the toll-like receptors (TLRs) that function as membrane-bound receptors on the cell membrane, leading to downstream transcriptional activation [5]. Consequently, this activates the nuclear factor κ-light-chain-enhancer of activated B cells (NF-κB) and induces the production of pro-inflammatory cytokines, leading to inflammation and apoptosis [1]. Furthermore, inflammatory cytokines generated in the intestine are not limited to the intestine and circulate around the body through mesenteric blood vessels and lymphatic vessels, causing inflammation [6]. In particular, when inflammatory cytokines circulating around the body move to the brain, they cause damage to the blood–brain barrier (BBB), which protects the brain from external antigens [7]. Thereafter, oxidative stress increases in the endothelial cells that make up the BBB, leading to apoptosis [8]. At that same time, inflammatory cytokines are released from the microglia, increasing the permeability of the BBB [9]. This increased permeability of the BBB causes neuroinflammation and the degeneration of various types of neurons, including cholinergic, adrenergic, and serotoninergic neurons, and contributes to cognitive dysfunction [8]. Additionally, a recent study has shown that short-chain fatty acids (SCFAs), which are metabolites of the gut microbiota, affect the integrity of the BBB in the gut–brain axis and are involved in the development of microglia and the regulation of inflammation [10]. However, IBD patients exhibit decreased short-chain fatty acid contents due to gut microbiota dysbiosis, which has been shown to contribute to cognitive dysfunction [11]. Ultimately, colitis caused by increasing intestinal permeability can cause inflammation and apoptosis in the brain, leading to cognitive dysfunction. Therefore, natural materials that can suppress intestinal inflammation are required to prevent the cognitive dysfunction caused by UC.

*Eucommia ulmoides* (*E. ulmoides*) is a perennial deciduous tree belonging to the *Eucommiaceae* family and the *Eucommia* genus and is widely used in Korea, China, and Japan [12]. The bark of *E. ulmoides* is used as an herbal medicine in oriental medicine to strengthen muscles and bones, while the leaves are mainly used as tea because they are effective in reducing cholesterol by promoting lipid metabolism [13]. It is known that *E. ulmoides* not only contains various bioactive compounds such as chlorogenic acid, caffeic acid, quercetin, and aucubin but also contains various amino acids and minerals [14]. Therefore, recent studies have reported that *E. ulmoides* not only shows anti-apoptotic effects by modulating oxidative stress but also exerts anti-inflammatory effects by reducing the secretion of inflammatory cytokines [3,15]. In particular, it has been reported that *E. ulmoides* leaves are effective in improving intestinal inflammation and damage to the intestinal epithelium in mice with dextran sulfate sodium (DSS)-induced colitis [16]. However, there is no research on the alleviation of cognitive dysfunction caused by DSS-induced colitis by administering *E. ulmoides* leaves. Therefore, this study was conducted to determine whether the aqueous extract of *E. ulmoides* leaves (AEEL) could alleviate cognitive dysfunction by regulating the JNK/TLR4 signaling pathway in DSS-induced colitis in mice.

## 2. Results

### 2.1. Identification of the Physiological Compound

**Figure 1:**
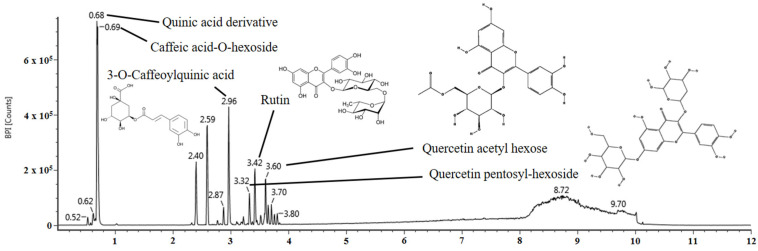
UPLC Q-TOF/MS^E^ chromatogram of the aqueous extract of *Eucommia ulmoides* leaves (AEEL).

The physiological compounds comprising the aqueous extract of *E. ulmoides* leaves (AEEL) were identified using UPLC Q-TOF/MS^E^ (Figure 1 and Table 1). The main fragments of the UPLC Q-TOF/MS^E^ chromatogram in negative ion mode were compared with those in previous studies. The MS^E^ spectra of UPLC Q-TOF/MS^E^ were identified as the following compounds: quinic acid derivative (retention time (RT), 0.68 min; fragments, 191, 109, and 85), caffeic acid-O-hexoside (RT, 0.69 min; fragments, 341, 179, 161, and 113), 3-O-caffeoylquinic acid (RT, 2.96 min; fragments, 191, 173, 161, and 85), quercetin pentosyl-hexoside (RT, 3.32 min; fragments, 301, 300, 271, and 255), rutin (RT, 3.42 min; fragments, 301, 300, 271, 255, and 151), and quercetin acetyl hexose (RT, 3.60 min; fragments, 301, 300, 271, 255, and 178.

### 2.2. Alleviative Effect of AEEL on DSS-Induced Symptoms

**Figure 2:**
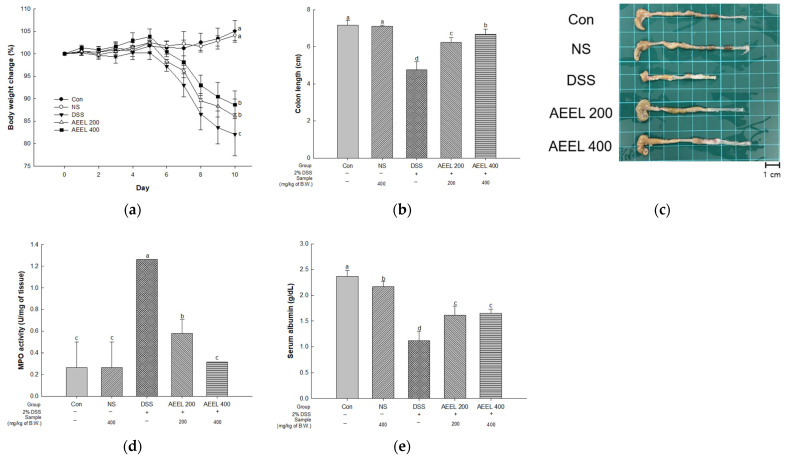
Alleviative effect of AEEL on DSS-induced colitis: bodyweight change (**a**), colon length (**b**), macroscopic appearance (**c**), myeloperoxidase (MPO) activity (**d**), and serum albumin contents (**e**). The results shown are mean ± SD (*n* = 6). The level of statistical significance of the data is *p* < 0.05, with different small letters indicating statistical differences between all groups.

To evaluate DSS-induced colitis symptoms in mice, the bodyweight change rate, colon length, myeloperoxidase (MPO) activity, and serum albumin contents were investigated (Figure 2). In terms of measuring the bodyweight change rate, there were no significant differences between the control (Con) group (105.00%) and the normal sample (NS) group (104.09%) (Figure 2a). However, the body weight of the DSS group’s (81.99%) significantly decreased compared to the Con group. Conversely, the body weight of the AEEL 200 group (86.13%) and the AEEL 400 group (88.62%) significantly increased compared to the DSS group.

Upon measuring the colon length, there were no significant differences between the Con group (7.16 cm) and the NS group (7.10 cm) (Figure 2b,c). However, the DSS group (4.76 cm) colon length significantly decreased compared to the Con group. Conversely, the colon lengths of the AEEL 200 group (6.28 cm) and the AEEL 400 group (6.66 cm) significantly increased compared to the DSS group.

Upon measuring the MPO activity, there were no significant differences between the Con group (0.26 U/mg) and the NS group (0.26 U/mg) (Figure 2d). However, the DSS group (1.26 U/mg) activity significantly decreased compared to the Con group. Conversely, the activity of the AEEL 200 group (0.57 U/mg) and the AEEL 400 group (0.31 U/mg) significantly increased compared to the DSS group.

Upon measuring serum albumin contents, the serum albumin contents of the DSS group (1.11 mg/dL) significantly decreased compared to the Con group (2.36 mg/dL) (Figure 2e). Conversely, the contents of the AEEL 200 group (1.61 mg/dL) and the AEEL 400 group (1.65 mg/dL) significantly increased compared to the DSS group.

### 2.3. Alleviative Effect of AEEL on Intestinal Permeability Dysfunction

**Figure 3:**
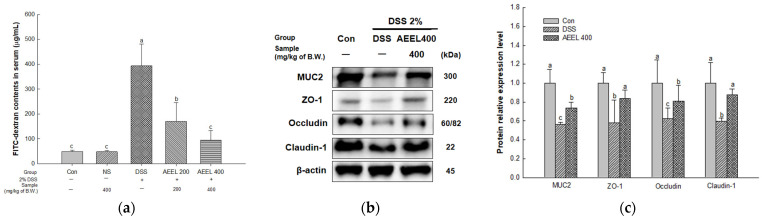
Alleviative effect of AEEL on DSS-induced intestinal permeability dysfunction. Fluorescein isothiocyanate (FITC)-dextran (**a**), band images of Western blot analysis (**b**), and the expression level of intestinal barrier-related signaling (**c**). Results shown are mean ± SD (FITC-dextran, *n* = 6; Western blot, *n* = 4). The level of statistical significance of the data is *p* < 0.05, with different small letters indicating statistical differences between all groups.

To evaluate intestinal permeability in DSS-induced mice, the fluorescein isothiocyanate (FITC)-dextran contents in serum and protein expression levels related to TJ, such as mucin 2 (MUC2), zonula occludens-1 (ZO-1), occludin, and claudin-1 in mouse colon tissues, were investigated (Figure 3). In terms of measuring FITC-dextran contents, there were no significant differences between the Con group (49.25 μg/mL) and the NS group (48.57 μg/mL) (Figure 3a). However, the DSS group (393.29 μg/mL) significantly decreased compared to the Con group. Conversely, the AEEL 200 group (170.72 μg/mL) and the AEEL 400 group (94.53 μg/mL) significantly increased compared to the DSS group.

The protein expression levels of MUC2 (0.56), ZO-1 (0.57), occludin (0.62), and claudin-1 (0.59) of the DSS group in colon tissues were significantly downregulated compared to the Con group (1.00) (Figure 3b,c). However, the AEEL 400 group (0.73, 0.83, 0.81, and 0.87, respectively) was significantly upregulated compared to the DSS group.

### 2.4. Animal Behavioral Tests

**Figure 4:**
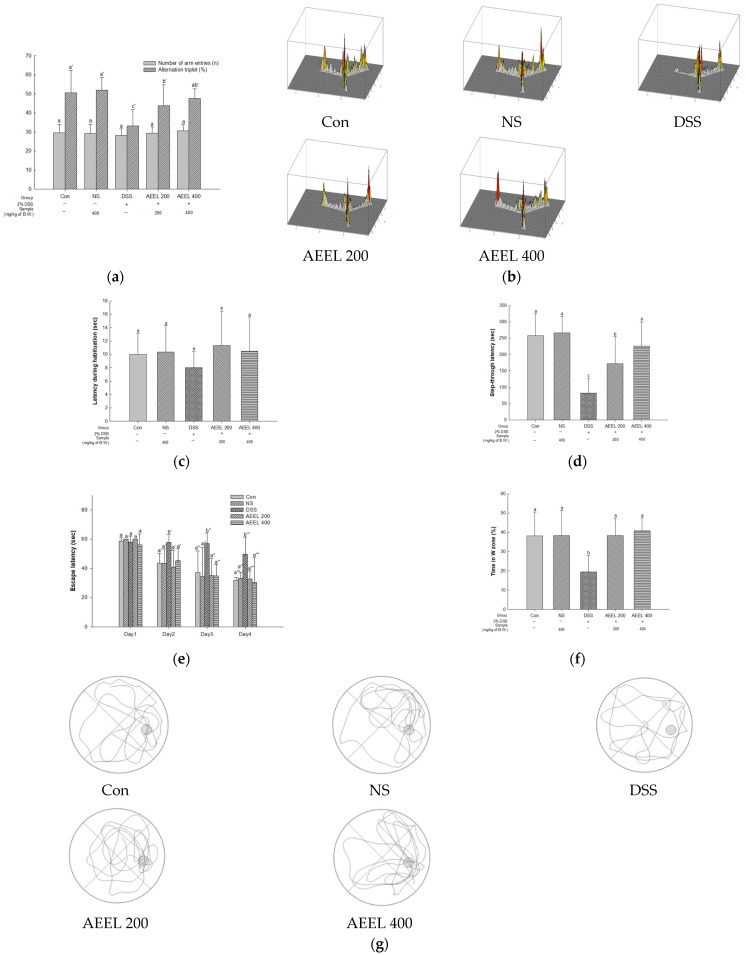
Alleviative effect of AEEL on DSS-induced learning and memory impairment. The number of arm entries and spontaneous alteration behavior (**a**) and the path tracing of each group (**b**) in the Y-maze test, latency during habituation (**c**) and step-through latency (**d**) in the passive avoidance test, and the escape latency (**e**), time in the W zone (**f**), and path tracing of each group (**g**) in the Morris water maze test. The results shown are mean ± SD (*n* = 6). The level of statistical significance of the data is *p* < 0.05, with different small letters indicating statistical differences. In the Y-maze test (**a**), a-c and a’-c’ mean the statistical difference for each experiment. In the Morris water maze test (**e**), a-b means the first day’s test; a’-b’ means the second day’s test; a”-b” means the third day’s test; a”’-b”’ means the fourth day’s test.

To evaluate cognitive function in DSS-induced mice, the Y-maze test, passive avoidance test, and Morris water maze test were conducted (Figure 4). Spontaneous alteration behavior was evaluated using the Y-maze test, and it was confirmed that there was no significant difference in the motility of the mice in all groups, as measured through the number of entries into each arm of the Y-shaped maze (Figure 4a). Additionally, there was no significant difference in alternative behavior between the Con group (50.67%) and the NS group (51.96%) (Figure 4a,b). However, the DSS group’s (33.16%) alternative behavior significantly decreased compared to that of the Con group. Conversely, the alternative behaviors of the AEEL 200 group (43.87%) and the AEEL 400 group (47.72%) were significantly increased compared to that of the DSS group.

Short-term memory function was evaluated through the passive avoidance test, and there was no significant difference in latency during habituation on the first day in all groups (Figure 4c). Afterward, there was no significant difference in the step-through latency between the Con group (258.16 s) and the NS group (260.33 s) on the second day (Figure 4d). However, that in the DSS group (82.83 s) significantly decreased compared to the Con group. Conversely, that in the AEEL 200 group (171.66 s) and the AEEL 400 group (226.00 s) significantly increased compared to the DSS group.

Long-term memory and spatial cognitive function were evaluated through the Morris water maze test, and there was no significant difference in escape latency in all groups on the first day (Figure 4e). On the fourth day, there was no significant difference in escape latency between the Con group (31.98 s) and the NS group (33.27 s). However, the DSS group (49.76 s) was significantly delayed compared to the Con group. Conversely, that of the AEEL 200 group (32.76 s) and the AEEL 400 group (30.37 s) latency significantly improved compared to the DSS group. In the probe tests, there was no significant difference in retention time between the Con group (38.12%) and the NS group (38.26%) (Figure 4f,g). However, the retention time of the DSS group (19.51%) significantly decreased compared to the Con group. Conversely, that of the AEEL 200 group (38.29) and the AEEL 400 group (40.74%) significantly increased compared to the DSS group.

### 2.5. SCFAs in Feces

The SCFA contents of the feces of DSS-induced mice are shown in Table 2. The contents of acetic acid (4.42 mM/g), propionic acid (4.65 mM/g), and butyric acid (1.69 mM/g) from the DSS group were significantly decreased compared to the Con group (8.04 mM/g, 4.79 mM/g, and 2.08 mM/g). However, the acetic acid and butyric acid contents of the AEEL 400 group (6.23 mM/g, 1.93 mM/g) were significantly increased compared to the DSS group.

### 2.6. Alleviative Effect of AEEL on Mitochondrial Dysfunction in the Brain

**Figure 5:**
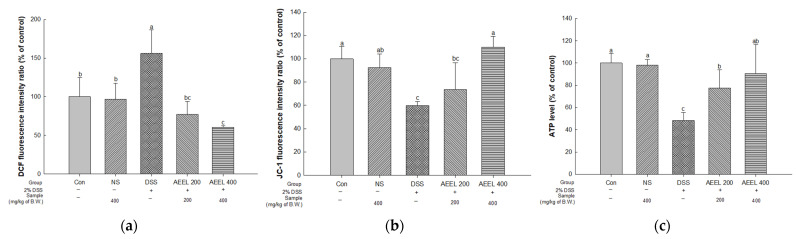
Alleviative effect of AEEL on DSS-induced mitochondrial dysfunction. Mitochondrial ROS level (**a**), mitochondrial membrane potential (**b**), and mitochondrial ATP level (**c**) in the brain. The results shown are mean ± SD (*n* = 3). The level of statistical significance of the data is *p* < 0.05, with different small letters indicating statistical differences between all groups.

To evaluate the mitochondrial function in DSS-induced mice, the mitochondrial reactive oxygen species (ROS), mitochondrial membrane potential (MMP), and adenosine triphosphate (ATP) in mouse brain tissues were investigated (Figure 5). Measuring the mitochondrial ROS levels, there were no significant differences between the Con group (100%) and the NS group (96.88% of the control) (Figure 5a). However, the DSS group (156.00% of the control) levels were significantly increased compared to the Con group. Conversely, the AEEL 200 group (77.37% of the control) and the AEEL 400 group (60.71% of the control) levels were significantly decreased compared to the DSS group.

Upon measuring the mitochondrial MMP, the DSS group result (59.77% of control) was significantly decreased compared to the Con group (100%) (Figure 5b). However, the AEEL 200 group (77.37% of the control) and the AEEL 400 group (60.71% of the control) results were significantly increased compared to the DSS group. In particular, there was no significant difference between the Con group and the AEEL 400 group.

Upon measuring mitochondrial ATP levels, there was no significant difference between the Con group (100%) and the NS group (98.26% of the control) (Figure 5c). However, the DSS group level (48.41% of the control) was significantly decreased compared to the Con group. Conversely, the AEEL 200 group (63.86% of control) and the AEEL 400 group (90.55% of control) levels were significantly increased compared to the Con group.

### 2.7. Alleviative Effect of AEEL on Cholinergic Dysfunction in Brain

**Figure 6:**
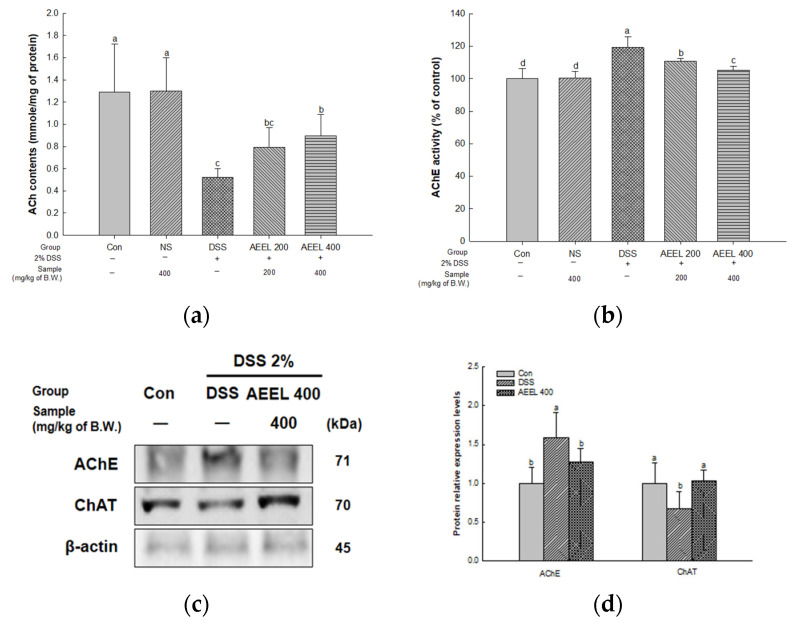
Alleviative effect of AEEL on DSS-induced cholinergic dysfunction. Acetylcholine (ACh) contents (**a**), acetylcholinesterase (AChE) (**b**), band images of Western blot analysis (**c**), and the expression level of cholinergic system signaling (**d**) in brain tissues. The results shown are mean ± SD (ACh contents and AChE activity, *n* = 6; Western blot, *n* = 4). The level of statistical significance of the data is *p* < 0.05, with different small letters indicating statistical differences between all groups.

To evaluate cholinergic function in DSS-induced mice, the acetylcholine (ACh) contents, acetylcholinesterase (AChE) activity, and protein expression levels related to the cholinergic system, such as the levels of AChE and choline acetyltransferase (ChAT) in mouse brain tissues, were investigated (Figure 6).

Upon measuring the ACh contents in brain tissues, there was no significant difference between the Con group (1.28 mmole/mg of protein) and the NS group (1.29 mmole/mg of protein) (Figure 6a). However, the DSS group content (0.52 mmole/mg of protein) was significantly decreased compared to the Con group. Conversely, the AEEL 200 group (0.79 mmole/mg of protein) and the AEEL 400 group (0.89 mmole/mg of protein) contents were significantly increased compared to the DSS group.

Upon measuring the AChE activity in brain tissues, there was no significant difference between the Con group (100%) and the NS group (100.37% of the control) (Figure 6b). However, the DSS group activity (119.32% of the control) was significantly increased compared to the Con group. Conversely, the activity of the AEEL 200 group (110.87% of the control) and the AEEL 400 group (105.38% of the control) was significantly decreased compared to the DSS group.

The protein expression level of AChE of the DSS group (1.58) in brain tissues was significantly upregulated compared to the Con group (1.00) (Figure 6c,d). However, the AEEL 400 group level (1.27) was significantly downregulated compared to the DSS group. The protein expression level of ChAT of the DSS group (0.67) was significantly downregulated compared to the Con group (1.00). However, the AEEL 400 group level (1.03) was significantly upregulated compared to the DSS group.

### 2.8. Alleviative Effect of AEEL on Oxidative Stress

**Figure 7:**
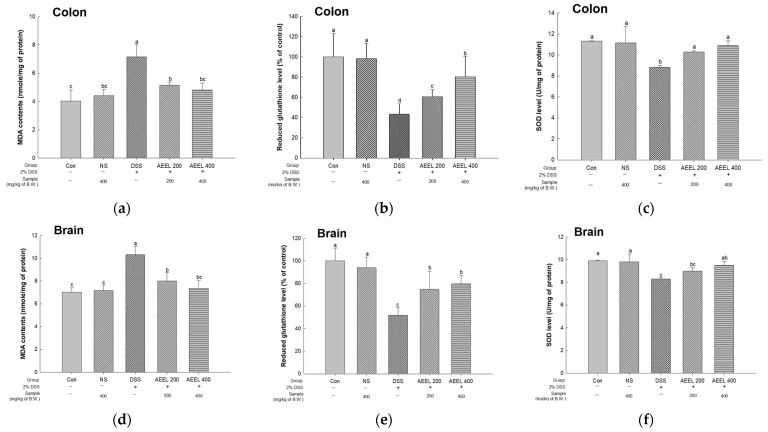
Alleviative effect of AEEL on DSS-induced oxidative stress. Malondialdehyde (MDA) contents, reduced glutathione (GSH) level, and superoxide dismutase (SOD) level in colon (**a**–**c**) and brain (**d**–**f**) tissues. The results shown are mean ± SD (*n* = 6). The level of statistical significance of the data is *p* < 0.05, with different small letters indicating statistical differences between all groups.

To evaluate oxidative stress in DSS-induced mice, the malondialdehyde (MDA) contents, reduced glutathione (GSH) level, and superoxide dismutase (SOD) level in colon and brain tissues were investigated (Figure 7). Of the results measuring the MDA contents in colon and brain tissues, the DSS group contents (colon, 7.16 nmole/mg of protein; brain, 10.32 nmole/mg of protein) were significantly increased compared to the Con group (colon, 4.03 nmole/mg of protein; brain, 7.02 nmole/mg of protein) (Figure 7a,d). However, the contents of the AEEL 200 group (colon, 5.16 nmole/mg of protein; brain, 8.01 nmole/mg of protein) and the AEEL 400 group (colon, 4.81 nmomle/mg of protein; brain, 7.36 nmole/mg of protein) were significantly decreased compared to the DSS group.

Upon measuring the reduced GSH levels in colon and brain tissues, there was no significant difference between the Con group (colon, 100% of the control; brain, 100% of the control) and the NS group (colon, 98.26% of the control; brain, 93.97% of the control) (Figure 7b,e). However, the DSS group contents (colon, 43.58% of the control; brain, 51.73% of the control) were significantly decreased compared to the Con group. Conversely, the contents of the AEEL 200 group (colon, 60.77% of the control; brain, 74.76% of the control) and the AEEL 400 group (colon, 80.39% of the control; brain, 79.68% of the control) were significantly increased compared to the DSS group.

Upon measuring the SOD levels in colon and brain tissues, there was no significant difference between the Con group (colon, 11.32 U/mg of protein; brain, 9.92 U/mg of protein) and the NS group (colon, 11.14 U/mg of protein; brain, 9.82 U/mg of protein) (Figure 7c,f). However, the DSS group levels (colon, 8.81 U/mg of protein; brain, 8.28 U/mg of protein) were significantly decreased compared to the Con group. Conversely, the levels of the AEEL 200 group (colon, 10.28 U/mg of protein; brain, 8.99 U/mg of protein) and the AEEL 400 group (colon, 10.89 U/mg of protein; brain, 9.50 U/mg of protein) were significantly increased compared to the DSS group.

### 2.9. Alleviative Effect of AEEL on Inflammation

**Figure 8:**
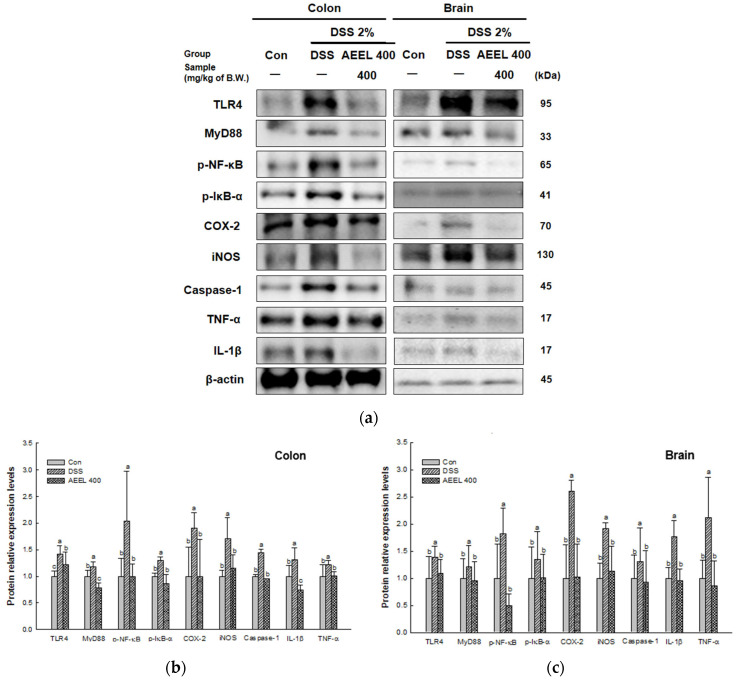
Alleviative effect of AEEL on DSS-induced inflammation response. Band images of Western blot analysis (**a**) in gut and brain tissues and the expression level of inflammation-related signaling in colon (**b**) and brain (**c**) tissues. The results shown are mean ± SD (*n* = 4). The level of statistical significance of the data is *p* < 0.05, with different small letters indicating statistical differences between all groups.

To evaluate the alleviative effect of AEEL in DSS-induced inflammation, the protein expression levels related to inflammation, such as toll-like receptor 4 (TLR4), myeloid differentiation primary response protein 88 (MyD88), the phosphor-nuclear factor κ-light-chain-enhancer of activated B cells (p-NF-κB), p-IκB-α, cyclooxygenase 2 (COX-2), inducible nitric oxide synthase (iNOS), cystein-aspartic acid protease 1 (caspase-1), interleukin-1β (IL-1β), and tumor necrosis factor α (TNF-α) in colon and brain tissues were investigated (Figure 8). The protein expression levels of TLR4 (colon, 1.41; brain, 1.38), MyD88 (colon, 1.17; brain, 1.21), p-NF-κB (colon, 2.03; brain, 1.81), p-IκB-α (colon, 1.30; brain, 1.34), COX-2 (colon, 1.90; brain, 2.60), iNOS (colon, 1.70; brain, 1.91), caspase-1 (colon, 1.43; brain, 1.30), IL-1β (colon, 1.31; brain, 1.76), and TNF-α (colon, 1.21; brain, 2.11) of the DSS group in colon and brain tissues were upregulated compared to the Con group (colon, 1.00; brain, 1.00). However, the AEEL 400 group levels (colon, 1.21, 0.78, 0.99, 0.85, 0.99, 1.15, 0.95, 0.74, and 1.00, respectively; brain, 1.09, 0.95, 0.49, 1.00, 1.02, 1.13, 0.92, 0.95, and 0.85, respectively) were downregulated compared to the DSS group.

### 2.10. Alleviative Effect of AEEL on Apoptosis

**Figure 9:**
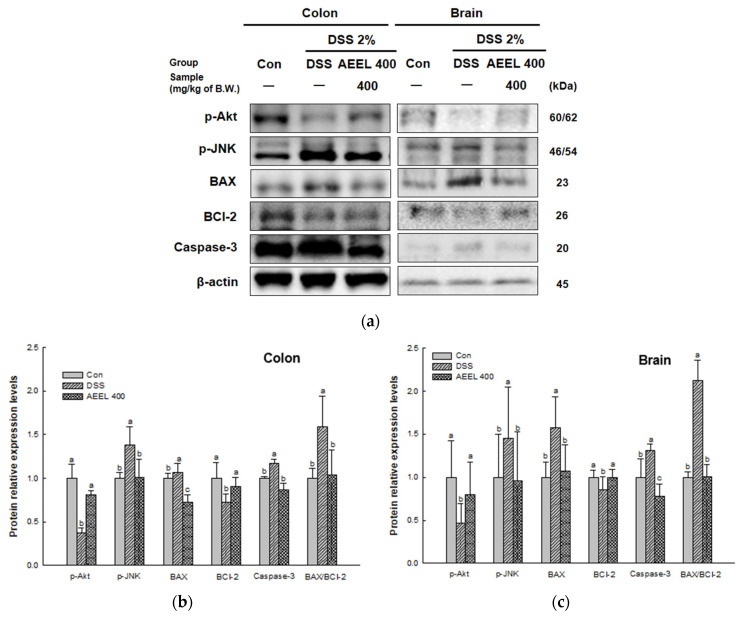
Alleviative effect of AEEL on the DSS-induced apoptosis response. Band images of Western blot analysis (**a**) and the expression level of apoptosis-related signaling in colon (**b**) and brain (**c**) tissues. The results shown are mean ± SD (*n* = 4). The level of statistical significance of the data is *p* < 0.05, with different small letters indicating statistical differences between all groups.

To evaluate the alleviative effect of AEEL in DSS-induced apoptosis, the protein expression levels related to apoptosis, such as those of phosphoprotein kinase B (p-Akt), phospho c-Jun-N-terminal kinase (p-JNK), BCl-2-associated X protein (BAX), B-cell lymphoma 2 (BCl-2), caspase-3, and the BAX/BCl-2 ratio were investigated (Figure 9). The protein expression levels of p-Akt (colon, 0.37; brain, 0.46) and BCl-2 (colon, 0.72; brain, 0.85) of the DSS group were downregulated compared to the Con group (colon, 1.00; brain, 1.00). However, the AEEL 400 group levels (colon, 0.81 and 0.90, respectively; brain, 0.80 and 1.00, respectively) were significantly upregulated compared to the DSS group. The protein expression levels of p-JNK (colon, 1.38; brain, 1.45), BAX (colon, 1.07; brain, 1.57), caspase-3 (colon, 1.17; brain, 1.31), and BAX/BCl-2 ratio (colon, 1.58; brain, 2.11) of the DSS group were upregulated compared to the Con group (colon, 1.00; brain, 1.00). However, the AEEL 400 group levels (colon, 1.00, 0.72, 0.87, and 1.04, respectively; brain, 0.95, 1.07, 0.77, and 1.00, respectively) were significantly downregulated compared to the DSS group.

## 3. Discussion

Recent studies have reported that the pathogenesis of IBD, such as UC and Crohn’s disease (CD), is associated with various systemic diseases, including liver and cardiovascular diseases, through systemic inflammation [6]. In particular, the development of neurodegenerative diseases such as Alzheimer’s disease (AD), which is characterized by systemic inflammation and neuronal apoptosis, has been reported to be associated with IBD [17]. Therefore, treatments have been developed to treat IBD, but it is known that it is difficult to completely cure the disease when it develops [17]. In addition, currently employed treatments such as 5-aminosalicylic acid (5-ASA), mesalamine, and sulfasalazine have been reported to have some side effects, including abdominal pain, nausea, headache, and fever [18]. Therefore, it is necessary to develop the use of natural materials with relatively few side effects for prevention and treatment.

Currently, it is now being reported that *E. ulmoides* leaves, a natural material with plant-derived bioactivity, can protect against a variety of diseases [14]. Therefore, prior to this study, UPLC Q-TOF/MS^E^ testing was performed to identify the various bioactive substances contained in AEEL. As a result, the bioactive compounds of AEEL were identified as quinic acid derivative, caffeic acid-O-hexoside, 3-O-caffeoylquinic acid, quercetin pentosyl-hexoside, rutin, and quercetin acetyl hexose (Figure 1 and Table 1). Consistent with this finding, previous studies reported that *E. ulmoides* leaves contain a variety of phenolics such as quinic acid, caffeic acid, quercetin, and rutin [14,19]. It was reported that *E. ulmoides* leaves contain 3-O-caffeoylquinic acid, which improved intestinal permeability by ameliorating IEC degeneration and necrosis in the colonic tissue from DSS-induced colitis in rats [20]. In addition, rutin administration ameliorated neurological disease by protecting neurons in 6-hydroxydopamine (6-OHDA)-induced Parkinson’s disease rats [21]. Therefore, this study was conducted to determine whether AEEL could alleviate cognitive dysfunction in mice with DSS-induced colitis.

Recent studies have reported that UC patients not only experience symptoms of intestinal inflammation but also show a decrease in body mass index (BMI) compared to non-sufferers [22]. This decrease in BMI suggests that IBD affects the body’s nutritional function, such as malnutrition and malabsorption, resulting in weight loss in the development of UC [23]. Therefore, we studied the effect of AEEL on improving colitis symptoms in mice with DSS-induced colitis. AEEL administration improved bodyweight change, colon shortening, MPO activity, and the albumin content of serum in mice with DSS-induced colitis (Figure 2). As in this study, Murakami et al. (2018) reported that *E. ulmoides* leaf extract improved weight loss, colon shortening, and MPO activity in mice with DSS-induced colitis [24]. According to Zhai et al. (2021), *E. ulmoides* leaf extract alleviated colon shortening and inflammation of the colon in mice with DSS-induced colitis [16]. These results confirm that AEEL intake can alleviate colitis symptoms in mice with DSS-induced colitis by alleviating body weight loss, colon length shortening, and inflammatory responses. Therefore, it is suggested that AEEL has potential value as a material that may improve IBD symptoms.

The gut barrier that protects the gut, which is the site of origin for IBD, is composed of the mucus layer, IECs, and TJs, which maintain the integrity of the barrier between the IECs and prevent external antigens from entering the intestine [25]. Activation of the intestinal immune system due to increased intestinal permeability, which can be caused by damage to the gut barrier due to complex factors such as genetic and environmental factors, is known to be the main mechanism for the development of UC [26]. Therefore, this study determined whether AEEL could alleviate intestinal permeability by improving TJ protein representation in the colon tissues of DSS-induced mice. As a result, AEEL improved intestinal permeability by maintaining the integrity of the gut barrier by upregulating the expression levels of TJ proteins such as mucin 2 (MUC2), zonula occludens-1 (ZO-1), occludin, and claudin-1 (Figure 3). According to Zhai et al. (2021), *E. ulmoides* leaves alleviated colon length shortening in DSS-induced mice, increased the levels of occludin and claudin-1, and were effective in improving the gut barrier [16]. In addition, according to Wan et al. (2021), *E. ulmoides* leaves containing 3-O-caffeoylquinic acid, known as chlorogenic acid, improved IEC degeneration and necrosis, neutrophil infiltration, etc., with hematoxylin eosin staining of the colon tissue of mice with colitis induced by DSS [20]. These results demonstrate that AEEL can effectively maintain intestinal barrier integrity in mice with DSS-induced colitis. Therefore, this suggests that AEEL could be used as a natural material to regulate intestinal inflammation by maintaining the intestinal barrier.

The development of UC has been reported to increase the risk of depression, mood disorders, and cognitive impairment, which, in turn, accelerates the development of neurodegenerative diseases, including Alzheimer’s disease and Parkinson’s disease (PD) [27]. He et al. (2021) reported that intestinal inflammation increased Aβ deposition, leading to neuroinflammation and spatial cognitive dysfunction in DSS-induced mice [28]. In addition, according to van Langenberg et al. (2017), short-term and long-term memory abilities and perceptual abilities were found to be decreased in IBD patients compared to healthy controls [29]. Therefore, it was confirmed by animal behavioral experiments such as Y-maze, passive avoidance, and Morris water maze tests that AEEL alleviates cognitive dysfunction in mice with DSS-induced colitis. As a result, AEEL was confirmed to have an alleviative effect on cognitive dysfunction in mice with DSS-induced colitis, with improvement effects on spontaneous alteration behavior, short-term memory, spatial cognitive ability, and long-term memory (Figure 4). According to Heitman et al. (2017), chlorogenic acid increased spontaneous alteration behavior in the Y-maze and alleviated memory impairment in the Morris water maze and the passive avoidance tests in scopolamine-induced mice [30]. Furthermore, according to Xing et al. (2019), *E. ulmoides* improved long-term memory and short-term memory performance in mice with Aβ-induced cognitive dysfunction in the passive avoidance test and Morris water maze test [14]. These results indicate that AEEL is effective in alleviating cognitive dysfunction that has been induced by DSS in mice. Therefore, these findings suggest that AEEL has the potential to alleviate behavioral disorders caused by colitis.

SCFAs are not only effective in maintaining intestinal barrier integrity in the gut but they are also known to have beneficial effects on nerve cell function and cognitive function [11]. In particular, SCFAs, which are produced by gut microbiota when metabolizing dietary fiber, are involved in the growth and differentiation of neurons and synapses in the CNS and are reported to play an important role in learning and memory [31,32]. Furthermore, they may prevent memory impairment by regulating the expression of neurotrophic factors such as glial cell line-derived neurotrophic factor (GDNF) and brain-derived neurotrophic factor (BDNF) [10]. Among them, acetic acid is the most abundant and is known to be converted to butyric acid by butyryl-CoA:acetate-CoA transferase [11]. In particular, butyric acid is effective in improving gut barrier function with the production of IECs, mucin, and TJs and the reduction of inflammation [32]. Additionally, it is known to reduce neuroinflammation in the brain and ameliorate BBB damage [33]. However, intestinal inflammation causes gut microbiota dysbiosis and reduces the abundance of SCFA-producing bacteria [11]. Therefore, we determined whether AEEL could ameliorate the decrease in SCFA contents in the feces of mice with DSS-induced colitis. As a result, AEEL was shown to alleviate the decrease in SCFA content induced by DSS in mice (Table 2). Zai et al. (2021) reported that chlorogenic acid significantly increased the butyric acid content in high-fat diet-induced rats [16]. In addition, Zhao et al. (2020) reported that *E. ulmoides* leaf extract significantly increased the contents of acetic acid and butyric acid in the feces of a senescence-accelerated mouse P6 (SAMP6) model [34]. Based on these results, it was found that AEEL alleviates the decrease in SCFA contents, helps to maintain intestinal barrier function, and alleviates cognitive dysfunction in DSS-induced mice. Therefore, this suggests that AEEL has the potential to protect gut barrier function and alleviate cognitive dysfunction by mitigating the decrease in SCFA contents.

When the inflammatory cytokines produced in the gut of IBD patients travel to the brain via systemic circulation, the inflammatory cytokines trigger oxidative stress and damage the mitochondria of brain nerve cells [35]. Mitochondria, which are organelles with a rod-shaped double membrane structure, generate energy in the form of ATP through the TCA cycle and electron transport chain under aerobic conditions [36]. Additionally, mitochondria play an important role in the nervous system because 20% of the ATP produced is consumed in the brain [37]. However, when mitochondrial dysfunction is induced due to oxidative stress, which is known to cause excessive reactive oxygen species (ROS) formation, mitochondrial dysfunction and acetyl-CoA production are reduced, and acetyl-CoA is released due to mitochondrial permeability transition pore (MPTP) open activation in the mitochondrial membrane, resulting in ATP deficiency [38]. Therefore, the alleviative effect of AEEL on mitochondrial function damage in mice induced by DSS was studied. As a result, AEEL was found to alleviate the ROS level in brain mitochondria and alleviate mitochondrial dysfunction by increasing MMP and ATP levels (Figure 5). Kong et al. (2019) reported that the chlorogenic acid contained in *E. ulmoides* leaves improved MMP and ATP levels in paraquat-induced A549 cells [39]. According to Kim et al. (2022), in PM_2.5_-induced mice, the ethyl acetate fraction of *E. ulmoides* leaves reduced ROS in mouse brain mitochondria and increased MMP and ATP levels [40]. These results demonstrate that AEEL can effectively ameliorate DSS-induced colitis mouse brain mitochondrial dysfunction. Thus, AEEL, which contains various bioactive substances, was shown to help maintain brain mitochondrial function in mice with DSS-induced colitis by improving ROS levels, MMP, and ATP levels.

The cognitive dysfunction seen in IBD patients is known to be due not only to mitochondrial damage but also to impaired cholinergic systems [41]. Cholinergic neurons in the brain synthesize neurotransmitters, such as ACh from acetyl-CoA and choline through ChAT in the presynaptic cytoplasm, and most cellular acetyl CoA is produced from pyruvate in the TCA cycle within mitochondria [42]. In addition, according to Wong-Guerra et al. (2017), mitochondrial dysfunction not only inhibits ACh synthesis in a mouse model of memory impairment but also increases AChE activity and contributes to ACh degradation [43]. In addition, it is reported that a deficiency in ACh causes cholinergic system dysfunction, in which signals are not transmitted between neurons, and AD, a representative degenerative brain disease that leads to cognitive dysfunction [35]. In this study, AEEL alleviated the decrease in ACh content in brain tissue and not only reduced AChE activity but also upregulated ChAT protein expression and downregulated AChE protein expression (Figure 6). According to Kim et al. (2022), in PM_2.5_-induced mice, the ethyl acetate fraction of *E. ulmoides* leaves alleviated the decrease in ACh content and reduced AChE activity [40]. According to Xing et al. (2019), *E. ulmoides* extract reduced AChE activity in the hippocampus and prefrontal cortex in a scopolamine-induced learning and memory impairment model [14]. In addition, it was reported that the extract showed effects in behavioral experiments by upregulating the expression level of cAMP response element-binding protein (CREB), which is related to synaptic plasticity, and BDNF, which is related to the development and maintenance of the central nervous system [44]. These findings indicate that AEEL alleviated the cognitive dysfunction caused by cholinergic dysfunction induced by DSS in the brains of mice. Therefore, this suggests that AEEL is a useful material to alleviate cognitive dysfunction in mice with DSS-induced colitis by modulating the cholinergic system.

Among the various immunomodulatory factors, oxidative stress has been proposed as one of the main mechanisms involved in the pathophysiology of IBD [45]. In general, ROS produced inside the body play an important role in the process of maintaining cellular homeostasis and regulating functions such as signaling and receptor activation [46]. However, excessive ROS react rapidly with biomolecules, causing an imbalance between oxidation and reduction in the body, resulting in oxidative stress, which causes lipid peroxidation, protein denaturation, and DNA damage [47]. Therefore, in order to control excessive ROS, the ROS level is reduced by various antioxidant systems in the body, including GSH, SOD, and catalase (CAT), but continuous oxidative stress disrupts the balance of the antioxidant system, reducing the ability to suppress oxidative stress [48]. Accordingly, it was confirmed whether the AEEL reduces the MDA level, a known indicator of oxidative stress, and increases the GSH and SOD levels, which are antioxidant enzymes in the body. The results showed that AEEL decreased MDA levels and increased GSH and SOD levels in brain and colon tissues (Figure 7). Gong et al. (2022) reported that *E. ulmoides* extract reduced the MDA level and increased the SOD and GSH levels in 150 mM HCl in 60% of EtOH-induced rats [49]. Shi et al. (2016) reported that the chlorogenic acid in *E. ulmoides* leaves decreased MDA levels and increased SOD, GSH, and CAT levels in the livers of rats with CCl_4_-induced liver fibrosis [50]. These results confirmed that AEEL can effectively regulate oxidative stress, inhibiting oxidative stress-induced mitochondrial dysfunction as well as mitigating the acceleration of inflammatory responses. This suggests that AEEL could be used as a natural material that can effectively regulate oxidative stress and contribute to alleviating the inflammation caused by oxidative stress.

Oxidative stress has been reported to be associated with inflammation and apoptosis [46]. Generally, in ulcerative colitis, environmental and genetic factors, as well as the proteases produced by intestinal microorganisms, damage the mucus layer, allowing external antigens to penetrate directly into the IECs [26]. When the IECs and TJs are damaged and gut barrier integrity is reduced, pattern recognition receptors (PRRs) such as TLRs on the cell membrane are activated to recognize external antigens such as lipoprotein, lipopolysaccharide (LPS), and RNA, thereby activating the immune system [1]. When an external antigen enters the cell through TLR4, which is known to recognize LPS among the TLRs, a signal is transmitted by TLR4 adapter proteins such as MyD88 to degrade IκB-α, which inhibits the activation of NF-κB [51]. Activated NF-κB then translocates to the nucleus and induces the expression of inflammatory factors such as iNOS, COX-2, TNF-α, and pro-IL-1β in the nucleus [52]. Afterwards, pro IL-1β is cleaved by caspase-1 and matured into IL-1β, then inflammation is induced by the mature IL-1β and TNF-α [53]. Additionally, when pro-inflammatory cytokines are produced in the intestines through this pathway and inflammation is induced, they circulate throughout the body through the blood vessels and lymph of the mesentery and cause systemic inflammation [6]. When the pro-inflammatory cytokines circulating throughout the body move to the brain, they damage the BBB and increase the permeability of the BBB, causing neuroinflammation through the TLR/NF-κB signaling pathway and increasing oxidative stress in brain neurons [54]. Therefore, inflammation-related factors were measured to determine whether AEEL could inhibit inflammation in brain and colon tissues. In the results, AEEL showed an improvement effect on inflammation by downregulating the expression levels of inflammation-related factors in DSS-induced mice (Figure 8). According to Kim et al. (2022), *E. ulmoides* extract downregulates the expression levels of inflammation-related factors such as TLR4 and caspase-1 in PM_2.5_-induced mice [40]. According to Zheng et al. (2022), chlorogenic acid, the main bioactive compound in *E. ulmoides* leaves, downregulated the expression levels of NF-κB, IL-1β, and TNF-α in a neonatal hypoxic-ischemic brain injury rat model [55]. These findings indicate that AEEL, which contains chlorogenic acid, can suppress inflammation by suppressing the expression of proteins related to inflammation. Therefore, this suggests that AEEL has the potential to reduce inflammation in the brain and alleviate cognitive dysfunction by modulating gut inflammation to suppress systemic inflammation.

Inflammation in the gut causes the infiltration of neutrophils into the intestinal mucosa, submucosa, and lamina propria, causing the excessive production of ROS in mitochondria [25]. Afterward, apoptosis occurs to maintain homeostasis by removing those cells damaged by inflammation and oxidative stress [56]. Apoptosis occurs when JNK activates BAX, an apoptotic factor, and BAX translocates from the cytoplasm to the mitochondrial outer membrane, where it induces the release of cytochrome C with the opening of MPTP [57]. When cytochrome C is released, apoptosis is induced by activating caspase 3, which is known to cause apoptosis [58]. Conversely, the activation of Akt inhibits the activation of JNK, while BCl-2 inhibits apoptosis by inhibiting the release of cytochrome C by BAX [59]. Therefore, apoptosis-related factors were measured to determine whether AEEL could inhibit apoptosis in brain and colon tissues. As a result, AEEL showed an improvement effect on apoptosis by downregulating the expression levels of apoptosis-related factors and upregulating the expression levels of anti-apoptosis-related factors in DSS-induced mice (Figure 9). According to Setyaningsih et al. (2022), chlorogenic acid inhibits apoptosis by decreasing the expression level of BAX and increasing the expression level of BCl-2 in streptozotocin (STZ)-induced rats [60]. Cheng et al. (2022) reported that *E. ulmoides* extract inhibits apoptosis by downregulating the expression level of BAX and cleaved caspase 3 and upregulating the expression level of BCl-2 in spinal cord-injury rats [61]. These findings indicate that AEEL can suppress apoptosis by suppressing the expression of proteins related to apoptosis in DSS-induced mice. In conclusion, AEEL, a natural material containing chlorogenic acid, has been shown to alleviate cognitive dysfunction in DSS-induced colitis models by maintaining intestinal barrier integrity and by inhibiting oxidative stress and inflammation-induced apoptosis.

## 4. Materials and Methods

### 4.1. Chemicals

DSS (molecular weight of 40 kDa) was purchased from Alfa Aesar (Haverhill, MA, USA). Formic acid, mannitol, bovine serum albumin (BSA), FITC-dextran (4 kDa), HEPES sodium salt, egtazic acid (EGTA), digitonin, KCl, KH_2_PO_4_, malate, HEPES, MgCl_2_, pyruvate, 5,5,6,6-tetrachloro-1,1,3,3-tetraethylbenzimidazolylcarbocyanine iodide (JC-1), ethylenediaminetetraacetic acid (EDTA), 1 mM ethylene glycol-bis (2-aminoethyl ether)-N, N, N′, N′-tetraacetic acid (EGTA), metaphosphoric acid, *o*-phthalaldehyde (OPT), Triton X-100, phenylmethane sulfonyl fluoride (pMSF), phosphoric acid, hydroxylamine, acetylthiocholine iodide (ATCI), 5,5′-dithiobis(2-nitrobenzoic acid) (DTNB), and all other chemicals used were purchased from the Sigma-Aldrich Chemical Co. (St. Louis, MO, USA).

### 4.2. Preparation of Aqueous Extracts of E. ulmoides Leaves (AEEL)

*E. ulmoides* leaves were purchased from Yeongcheon ( Republic of Korea) and verified by the National Institute of Forest Science (Suwon, Republic of Korea). The sample was dried with hot air at 40 °C and then ground. After that, the powdered sample was extracted with distilled water for 2 h at 40 °C and filtered through a No. 2 filter paper (Whatman plc, Kent, UK). The filtered extract was evaporated using a rotary vacuum evaporator (N-N series, Eyela Co., Tokyo, Japan). The resulting aqueous extract of *E. ulmoides leaves* (AEEL) was lyophilized and stored at −20 °C until use.

### 4.3. UPLC Q-TOF/MS^E^

AEEL was dissolved in 50% MeOH and used for analysis. The major bioactivity compound in AEEL was identified using a Water Acquity ultraperformance liquid chromatography quadrupole time-of-flight mass spectrometry (UPLC Q-TOF/MS^E^) device. For separation of the compound, we used a 2.1 × 100 nm × 1.7 μm ACQUITY UPLC BEH C18 column (Waters Corp., Milford, MA, USA) in negative mode. The solvent gradient of mobile phase (solvent A: 0.1% formic acid in distilled water, solvent B: 0.1% formic acid in acetonitrile) was conducted as follows, with an oven temperature of 40 °C and a flow rate of 0.35 mL/min: 0–8 min B: 0–40%; 8–9 min B: 40–100%; 10–10.20 min B: 100–0%; 10.20–12 min B: 0%. The analysis conditions of MSE were lamp collision energy at 10–30 eV, fragmented voltage at 175 V, capillary voltage at 3 kV, source temperature at 100 °C, and a mass range from 50 to 1500 m/z [40].

### 4.4. Animal Experiment Designs

**Figure 10:**
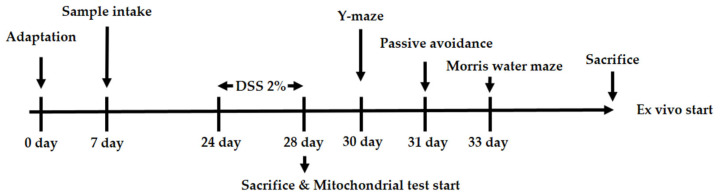
Experimental design for the in vivo and ex vivo tests of the DSS-induced mice model.

C57BL/6 mice (male, 4 weeks) were obtained from Samtako Inc. (Osan, Republic of Korea). Mice were bred under set conditions as follows: constant temperature (21–23 °C), humidity (50–55%), and a 12 h light/dark cycle. In addition, the mice were placed in cages, with either 3 or 2 mice per cage. After adaptation for 1 week, each group (*n* = 10) was randomly divided into 5 groups: the Con group, NS (AEEL 400 mg/kg of body weight (B.W.)) group, DSS group, DSS + AEEL 200 group (AEEL 200 mg/kg of B.W.), and DSS + AEEL 400 group (AEEL 400 mg/kg of B.W.). Extract from *Eucommia ulmoides* leaves was dissolved in drinking water and administered orally via a stomach tube once a day for 3 weeks. Thereafter, except for the Con and NS groups, the groups were fed 2% (*w*/*v*) DSS dissolved in drinking water for 5 days. Additionally, the 2% (*w/v*) DSS was replaced every 2 days. All animal experimental procedures were conducted with the approval of the Institutional Animal Care and Use Committee (IACUC) of Gyeongsang National University (Certificate No. GNU-221011-M0120, approved on 11 October 2022). During the above experiment, the body weight of the mice was measured for the final 11 days and the colon length was measured after the mice were dissected. The experimental design presented in Figure 10.

### 4.5. MPO Activity in the Colon 

The colon tissues harvested from the mice were homogenized with 50 mM phosphate buffer (pH 6.0) containing 0.5% hexadecyltrimethylammonium bromide (HTAB) and centrifuged at 4 °C for 20 min at 10,000× *g*. The supernatants, 0.0005% H_2_O_2_, and 50 mM potassium phosphate buffer (pH 6.0) with o-dianisidine dihydrochloride were mixed. The mixture was analyzed at 450 nm with a microplate reader (Epoch 2, BioTek, Winooski, VT, USA). MPO activity was detected in units (U) of MPO/mg tissue, assuming that 1 unit was defined as the amount required to disassemble 1 μmol of H_2_O_2/_min [61].

### 4.6. Albumin Measurement in Serum 

Blood was collected from the abdominal veins of the mice and stored in a heparin tube. The collected blood was centrifuged at 4° C for 10 min at 13,000× *g*. After that, the serum was analyzed for albumin levels using a biochemical analyzer (Fuji Dri-chem 4000i; Film Co., Tokyo, Japan).

### 4.7. Fluorescein Isothiocyanate (FITC)-Dextran Contents in Serum

To assess intestinal permeability, after fasting for 6 h, the mice were orally administered 60 mg/mL FITC-dextran solution (dose 0.4 mg/g B.W.). Blood was collected from the abdominal veins of the mice. The collected blood was centrifuged at 4 °C for 10 min at 13,000× *g*. The serum was analyzed using a fluorometer with an excitation wave of 485 nm and an emission wave of 535 nm (Infinite 200, Tecan Co., Männedorf, Switzerland). The FITC-dextran contents in serum were analyzed using a standard curve serially diluted in PBS [61].

### 4.8. Animal Behavioral Tests

#### 4.8.1. Y-maze Test

The Y-maze was designed with a black plastic three-armed structure (33 × 15 × 10 cm). Each arm of the maze was divided into areas A, B, and C and the mouse was placed at the end of each arm. After allowing the mouse to move freely in the maze for 8 min, movement and path tracing were recorded using a video tracking system (Smart 3.0, Panlab, Barcelona, Spain) [62].

#### 4.8.2. Passive Avoidance Test

The passive avoidance equipment consisted of a dark room that had electric shock capability and a bright room and had a gateway to move to each room. On the first day, the mouse was placed in a bright room without light and allowed to adapt for 1 min. Afterward, the light was turned on the bright room, and the mouse was allowed to adapt for 2 min. After that, when the gateway was opened, the mouse entered the dark room on all fours, it received an electric shock at 0.5 mA for 3 s, and the latency was recorded. On the second day, using the same method as on the first day, the step-through latency time to re-enter the dark room was set to be up to 300 s [63].

#### 4.8.3. Morris Water Maze Test

The Morris water maze was designed as a circular pool (90 cm in diameter and 30 cm in height); this was filled with water and the temperature was maintained at 23 ± 2 °C. The pool was randomly divided into 4 zones (N, E, S, and W) based on the zones marked on the outside of the pool. The platform in the W zone was placed below the water level and the pool water was filled with non-toxic white tempera paint. The mouse was allowed to swim freely to find the platform on its own for 1 min and was trained repeatedly for 4 days. After that, the platform was removed, and the retention time in the W zone was detected using the video tracing software (Smart 3.0, Panlab) for 1 min [63].

### 4.9. Determination of SCFAs in Feces

The feces of mice were mixed with 5 mM NaOH to homogenize them and were then centrifuged at 12,000× *g* for 10 min at 4 °C. The supernatants were mixed with propyl chloroformate and propanol/pyridine solution (*v*/*v* = 3:2). The stabilized mixture was mixed with hexane and centrifuged at 4 °C for 10 min at 15,000× *g*. The hexane layer was used to determine the SCFA level using an Agilent 7890A gas chromatograph (Agilent, Santa Clara, CA, USA) and DB-5MS column (thickness, 0.25 μM; length, 30 m; diameter, 0.25 mm; Agilent). The conditions were set as follows: injection temperature, 260 °C; split ratio, 50:1; flow rate, 1.0 mL/min; column oven temp, 40 °C; carrier gas, helium. The total program was completed in 16.5 min.

### 4.10. Assessment of Mitochondrial Function in the Brain

#### 4.10.1. Extraction of Mitochondria

Brain tissues harvested from mice were homogenized with mitochondrial isolation solution (75 mM sucrose, 215 mM mannitol, 0.1% bovine serum albumin (BSA), 20 mM HEPES, and 1 mM EGTA). The homogenates were centrifuged at 4 °C for 5 min at 1500× *g* and the supernatants were centrifuged at 4 °C for 15 min at 12,000× *g*. After that, the pellet that removed the supernatant was mixed with the isolation solution containing 0.1% digitonin in DMSO and left on ice for 5 min. The mixture was added to an isolation solution containing 1 mM EGTA and then centrifuged at 4 °C for 10 min at 15,000× *g* [64].

#### 4.10.2. Mitochondrial ROS Level

The mitochondrial extracts were mixed with 25 μM DCF-DA in respiration buffer (125 mM potassium chloride, 20 mM HEPES, 5 mM pyruvate, 2.5 mM malate, 2 mM potassium phosphate monobasic, 1 mM magnesium chloride, and 500 μM EGTA) and incubated for 20 min. The mixture was analyzed with a fluorometer set at an excitation wave of 485 nm and an emission wave of 535 nm (Infinite 200, Tecan Co.) [64].

#### 4.10.3. MMP

The mitochondrial extracts were mixed with JC-1 in an isolation solution containing 5 mM malate and 5 mM pyruvate and were then left for 15 min in a dark room. After that, the mixtures were analyzed on a fluorometer set at an excitation wave of 535 nm and an emission wave of 590 nm (Infinite 200, Tecan Co.) [64].

#### 4.10.4. Mitochondrial ATP Level

After measuring ROS and MMP, the remaining mitochondrial extracts were centrifuged at 4 °C for 10 min at 10,000× *g*. After that, the pellet used to remove the supernatant was mixed with 1% TCA, left on ice for 10 min, and then mixed with 25 mM tris-acetate buffer (pH 7.7). The mixture was centrifuged at 4 °C for 10 min at 12,000× *g*, then the supernatant was analyzed using an ATP kit with a luminometer (GloMax-Multi Detection System, Promega Co., Madison, WI, USA).

### 4.11. Cholinergic System

#### 4.11.1. ACh Contents

Brain tissue harvested from mice was homogenized with PBS and centrifuged at 4 °C for 25 min at 13,000× *g*. The supernatant was mixed with an alkaline hydroxylamine reagent (3.5 N NaOH and 2 M hydroxylamine in 1 M HCl) for 1 min at room temperature. The mixture was reacted with 0.37 M FeCl_3_·6H_2_O dissolved in 0.1 N HCl and 0.5 N HCl. The reactant was analyzed at 540 with a microplate reader (Epoch 2, BioTek) [40].

#### 4.11.2. AChE Activity

The supernatant used to measure ACh contents was mixed with 50 mM of sodium phosphate buffer and incubated at 37 °C for 15 min. The mixture was reacted with Ellman’s reaction solution (500 μM acetylthiocholine in 1 mM 5,5-dithiobis-(2-nitrobenzoic acid)) and incubated at 37 °C for 5 min. The reactant was then analyzed at 405 nm with a microplate reader (Epoch 2, BioTek) [40].

### 4.12. Antioxidant Activity

#### 4.12.1. MDA Contents

Colon and brain tissues harvested from the mice were homogenized with PBS and centrifuged at 2500× *g* for 10 min at 4 °C. The supernatants were mixed with 0.67% thiobarbituric acid and 1% phosphoric acid for 1 h in a 95 °C water bath. The mixtures were centrifuged at 4 °C for 10 min at 600× *g*, then the supernatants were analyzed at 530 nm (UV-1800, Shimadzu, Tokyo, Japan) [65].

#### 4.12.2. Reduced GSH Level

Colon and brain tissues harvested from the mice were homogenized with sodium phosphate buffer (pH 6.0) and centrifuged at 4 °C for 10 min at 15,000× *g*. The supernatants were mixed with 5% metaphosphoric acid and centrifuged at 2000× *g* for 2 min at 4 °C. The supernatants were mixed with 1 mg/mL o-phthaldialdehyde, 0.65 N NaOH, and 0.26 M Tris-HCl (pH 7.8) and incubated for 15 min at room temperature. The mixtures were analyzed using a fluorometer set at an excitation wave of 320 nm and an emission wave of 420 nm (Infinite 200, Tecan Co.) [65].

#### 4.12.3. SOD Level

Colon and brain tissue harvested from the mice were homogenized with PBS and centrifuged at 4 °C for 10 min at 400× *g*. The pellet, which removed the supernatants, was mixed with 1× cell extraction buffer containing 10% SOD buffer, 0.4% Triton X-100, and 200 μM phenylmethane sulfonylfluoride and then left on ice for 30 min. The mixtures were centrifuged for 10 min at 4 °C at 10,000× *g* and the supernatants were analyzed using a SOD kit (Dojindo Molecular Technologies) at 450 nm with a microplate reader (Epoch 2, BioTek).

### 4.13. Western Blot Assay

Colon and brain tissues harvested from the mice were homogenized with lysis buffer (GeneAll Biotechnology, Seoul, Republic of Korea) containing a 1% protease inhibitor (Quartett, Berlin, Germany). The homogenates were centrifuged at 13,000× *g* for 10 min at 4 °C and the proteins of the supernatant were separated by sodium dodecyl sulfate polyacrylamide gel electrophoresis (SDS-PAGE). Thereafter, the gels were transferred onto a polyvinylidene fluoride (PVDF) membrane (Millipore, Billerica, MA, USA). The membranes were blocked with 5% skim milk for 1 h at room temperature and reacted with a primary antibody for 12 h at 4 °C. The reacted membranes were then reacted with a secondary antibody for 1 h at room temperature and detected with a Chemidoc (iBright CL1500, Invitrogen, Carlsbad, CA, USA). The intensity of the band on the membrane image was calculated using ImageJ software (National Institutes of Health, Bethesda, MD, USA).

### 4.14. Statistical Analysis

All experimental results were expressed as the mean ± standard deviation (SD). The significance of the experimental results was analyzed by a one-way analysis of variance and significant differences among each group were verified at the 5% level by Duncan’s multiple range test, using the SAS software 9.4 (SAS Institute, Cary, NC, USA).

## 5. Conclusions

In summary, DSS-induced colitis caused cognitive dysfunction as well as intestinal inflammation. However, the administration of AEEL with various phenolic compounds maintained intestinal barrier integrity by regulating the TJ proteins. By maintaining intestinal barrier integrity, AEEL reduced colitis symptoms such as weight change, colon shortening, and hypoalbuminemia. In addition, AEEL alleviated cognitive dysfunction by improving mitochondrial function and the cholinergic system in brain tissue. Finally, AEEL effectively inhibited apoptosis by alleviating oxidative stress and reducing inflammatory responses by modulating the TLR4/NF-κB signaling pathway in intestinal and brain tissues. Ultimately, our results suggest that AEEL could be used as a natural material with the potential to suppress colitis-induced systemic inflammation by regulating the JNK/TLR4 signaling pathway and could alleviate cognitive dysfunction in the brain.

## Figures and Tables

**Table 1:** Physiological compounds identified in AEEL.

No.	Retention Time (min)	Parent Ion (*m/z*)	Fragment Ion (*m/z*)	Compound
1	0.68	533	191, 109, 85	Quinic acid derivative
2	0.69	683	341,179, 161, 113	Caffeic acid-O-hexoside
3	2.96	353	191, 173, 161, 85	3-O-Caffeoylquinic acid
4	3.32	595	301, 300, 271, 255	Quercetin pentosyl-hexoside
5	3.42	609	301, 300, 271, 255, 151	Rutin
6	3.60	505	301, 300, 271, 255, 178	Quercetin acetyl hexose

**Table 2:** Analysis of fecal short-chain fatty acids (SCFAs) on mice with DSS-induced colitis (unit: mM/g).

	Acetic Acid	Propionic Acid	Butyric Acid
Con	8.04 ± 0.09 ^a^	4.79 ± 0.10 ^a^	2.08 ± 0.09 ^a^
DSS	4.42 ± 0.42 ^c^	4.65 ± 0.08 ^b^	1.69 ± 0.18 ^c^
AEEL 400	6.23 ± 0.65 ^b^	4.65 ± 0.01 ^b^	1.93 ± 0.12 ^b^

The results shown are mean ± SD (*n* = 3). The level of statistical significance of the data is *p* < 0.05, with different small letters indicating statistical differences between all groups.

## Data Availability

The data presented in this study are available on request from the corresponding author as applicable.
